# Robust markers associated with floral traits in roses are suitable for marker-assisted selection across gene pools

**DOI:** 10.1007/s11032-023-01438-5

**Published:** 2023-12-08

**Authors:** Dietmar Schulz, Marcus Linde, Thomas Debener

**Affiliations:** 1 Institute of Plant Genetics, Molecular Plant Breeding Section, Leibniz University Hannover, Herrenhäuser Straße 2, 30419 Hannover, Germany; 2 Bundesamt Für Verbraucherschutz Und Lebensmittelsicherheit, Referat 231/Abteilung 2, Bundesallee 51, 38116 Brunswick, Germany

**Keywords:** SNP, KASP, MAS, Rosa, GWAS

## Abstract

We investigated the potential of markers associated with floral traits for parental selection in a cut rose breeding program. We analysed six Kompetitive Allele Specific PCR (KASP) markers for three important floral traits, petal length, petal number and scent, derived from experiments in a garden rose population. The six markers were applied to genotype a collection of 384 parental genotypes used for commercial cut rose breeding. We phenotyped a selection of progeny derived from pairs of parents having either high or low dosages of (contrasting) marker alleles associated with these traits. Significant differences were found between the contrasting progeny groups for each of the traits, although parents with the optimal allele dosage combinations could not always be used for the crosses. This not only supports the robustness of these marker‒trait associations but also demonstrates their potential for commercial rose breeding. It also demonstrates the use of marker information generated in garden rose populations for cut rose breeding.

**Supplementary Information:**

The online version contains supplementary material available at 10.1007/s11032-023-01438-5.

## Introduction

The cultivation of roses probably began approximately 5000 years ago in North Africa, West Asia and China for a variety of uses, including medicine, essential oils and food. Rose breeding with controlled crosses has been reported since the beginning of the nineteenth century (Leroy et al. 2023). Today, the breeding objectives for garden, cut and pot roses are different. Whereas transportability, productivity and vase life are very important for cut roses, disease resistance and cold and heat tolerance are the main breeding goals for garden roses. Several authors have noted that there are differences between the gene pools used for breeding garden, pot and cut roses (De Cock et al. 2007; Gudin 2003; Leus et al. 2018). The generation of new genetic variability through large numbers of controlled hand crosses is the first step in breeding highly heterozygous and mostly tetraploid, clonally propagated rose cultivars. Currently, parental genotypes are mainly selected for their fertility and hip production and for their harmonising ornamental and agronomic traits (Crespel and Mouchotte 2003). The first negative selection at the seedling stage is made for highly heritable and easily evaluated floral traits on a single plant per genotype in family sizes of usually less than 150 progeny from a single cross (Chaanin 2003). In the larger German breeding companies, this reduces the enormous number of 160,000 to 500,000 seedlings for cut and garden roses to 5–10% after the first season of selection. In other European breeding companies, the number of seedlings is significantly lower, and the selection is even stronger (Leus et al. 2018). In the following 3–4 years (cut roses) or 7–9 years (garden roses), selection is carried out on an increasing number of clones per genotype, resulting in approximately 15–23 cultivars introduced to the market per year and breeder. This means that only approximately 0.01% of the original number of seedlings is selected for their superior new flower characteristics or habit, with the highest selection intensity at the seedling stage.

Marker-assisted selection (MAS) has become a standard practice in major crops such as cereals and sugar beet (Lenaerts et al. 2019; Salgotra and Stewart 2020). The potential parental plants, and often large numbers of progeny, are evaluated using markers for the presence of selected resistance alleles, their hybrid status or other important agronomic traits. Given current commercial rose breeding practices, the genotyping of a large number of progeny would be difficult to adapt, but the selection of superior parents may be a suitable option. The selection of parents with higher allele dosages for major loci controlling important traits could significantly increase the proportion of progeny with the desired phenotypes (Smulders et al. 2019). This could make it possible to greatly reduce the number of crosses and seedlings while retaining enough improved seedlings to perform selection on additional traits between them and/or increase the selection intensity for these traits.

In this study, we used six validated SNP markers previously discovered in Genome Wide Association Study (GWAS) on only 95 garden rose genotypes to select superior parental combinations of cut roses. We were able to show that MAS of parental genotypes significantly increased the proportion of desired phenotypes in their progeny for three traits of major interest in cut rose breeding, despite the supposed differences between the two gene pools of cut and garden roses.

## Materials and methods

### Plant material

The association panel used to develop the six SNP markers presented here consisted of 95 mostly tetraploid rose cultivars from different breeding companies. This panel and the independent population used for marker validation are described in Schulz et al. (2016, 2021). A total of 384 cut rose genotypes were provided by Rosen Tantau KG and screened with six SNP markers.

Based on the SNP data, controlled hand crosses were performed for the selected genotypes, aiming for high or low petal length, high or low petal number and strong or weak fragrance. All plants were grown under standard semi-controlled greenhouse conditions. Only 15 of the 50 progenies produced had more than 10 surviving individuals and were subsequently used for phenotyping petal traits.

### Phenotyping for petal length

**Fig. 1:**
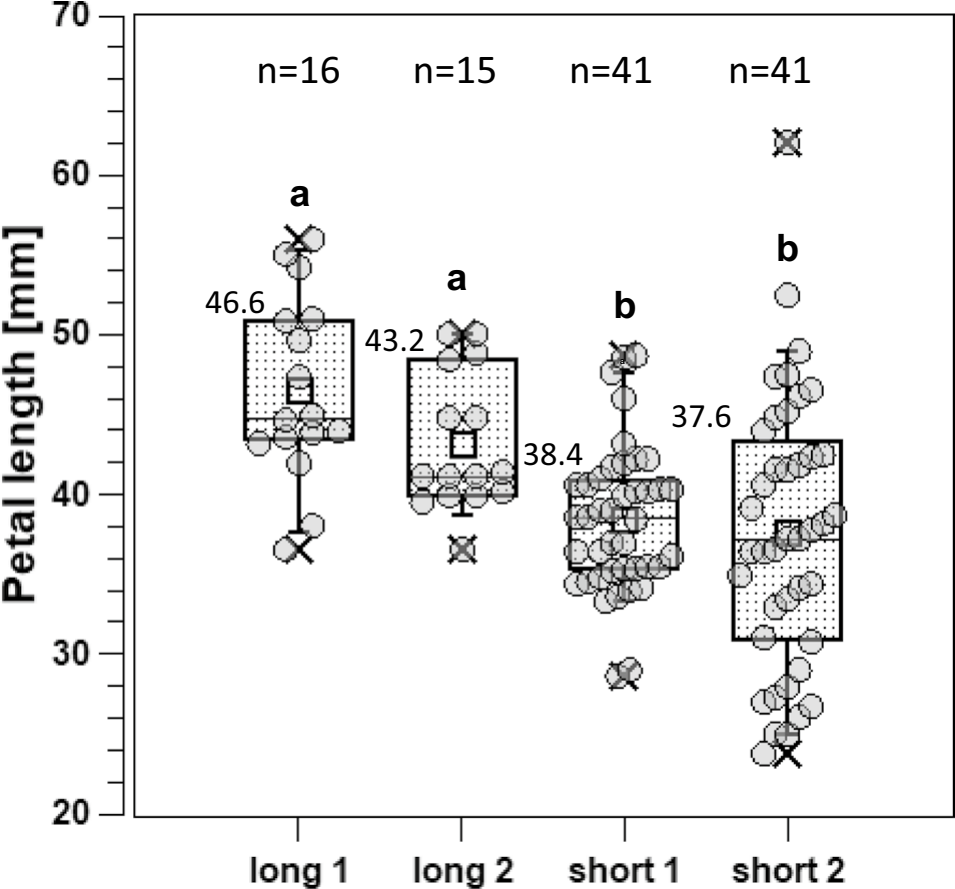
Box plots of four progenies analysed for petal length. On the left are the two progenies with long petals (long 1 and long 2), and on the right are the two progenies with short petals (short 1 and short 2). Significantly different means (two-sample *t* test; *p* ≤ 0.05) are indicated by different letters above the whiskers. For each progeny, the number of individuals (*n*) is given; mean values are shown to the left above the box

The length of the five outermost petals of the single flower of each seedling was measured in millimetres on graph paper. The arithmetic mean was calculated from the five values of each progeny and used as the value for that genotype. The distribution of the petal length is shown in the box plots for the four segregating progenies in Fig. 1.

### Phenotyping for petal number

**Fig. 2:**
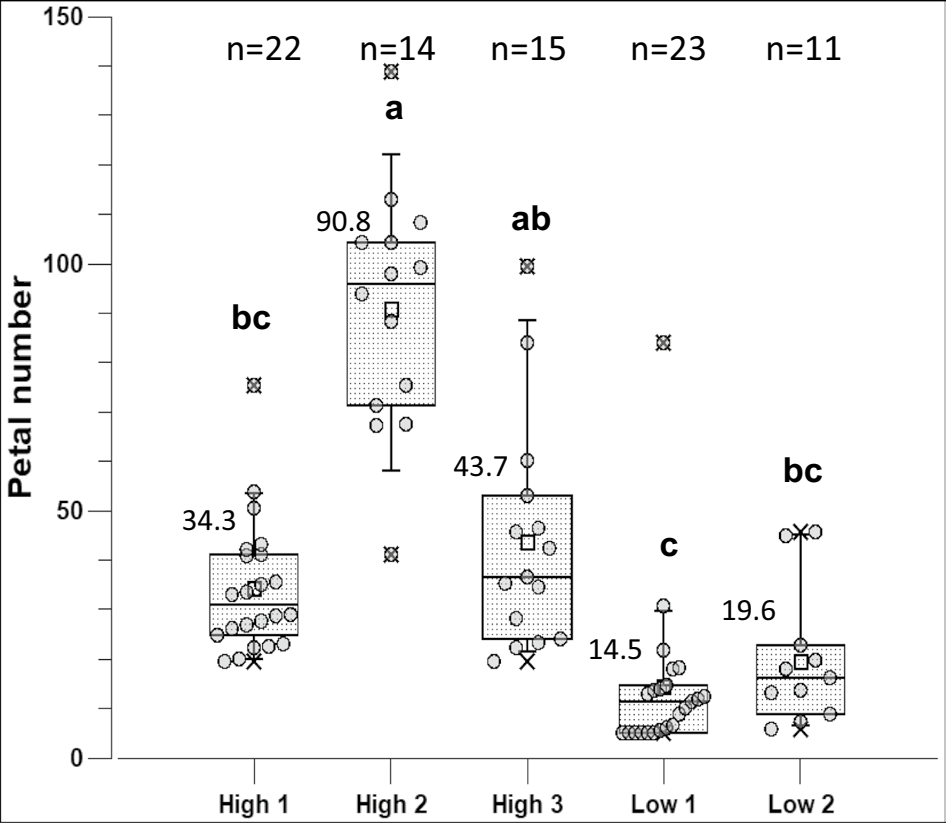
Box plots for mean petal number of progeny from five crosses with contrasting allele dosages of markers associated with petal number. On the left are the three high-petal-number progeny (high 1 to high 3), and on the right are the two low-petal-number progeny (low 1 and low 2). The number of individuals scored per group is given above the box plots. The means of two groups are significantly different when different letters are shown above the boxes (Kruskal‒Wallis analysis of variance, Dunn’s post hoc test; *p* ≤ 0.05). For each progeny, the number of individuals (*n*) is given; mean values are shown to the left above the box

The number of petals of the single flower of each seedling was counted. The arithmetic mean and median were calculated from the values of the individuals from the low-petal-number crosses and the high-petal-number genotypes. The mean petal number was used as the value for a particular genotype. The distribution of the petal number is shown in the box plots for the five segregating progenies in Fig. 2.

### Phenotyping for fragrance

**Fig. 3:**
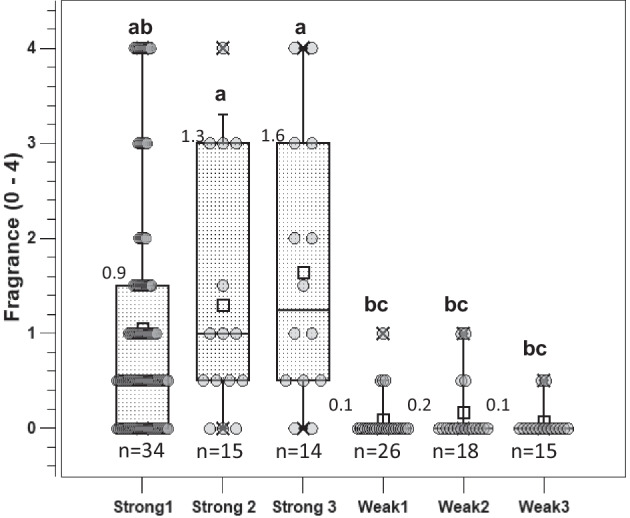
Box plots for median fragrance scores of progeny from five crosses with contrasting allele dosages for the fragrance-associated markers. On the left are the three progeny for strong fragrance (strong 1 to strong 3), and on the right are the two progeny for weak fragrance (weak 1 to weak 2). The number of individuals scored per group is given above the box plots. Significant differences between the means of two groups are indicated by different letters above the boxes (Kruskal‒Wallis analysis of variance, Dunn’s post hoc test; *p* ≤ 0.05). For each progeny, the number of individuals (*n*) is given; mean values are shown on the left side above the box

Fragrance was assessed manually by three people by simply smelling a harvested and bagged single flower from each seedling. The fragrance was graded according to five classes: 0 = no fragrance, 1 = mild to moderate fragrance, 2 = good fragrance, 3 = strong fragrance and 4 = very strong fragrance. The final median value obtained resulted from individual testing conducted by three persons on each flower of every genotype. To ensure the same scoring of the fragrance as in Schulz et al. (2021), the flowers of the cultivars Black Baccara (0 = no fragrance), Elfe (1 = mild to moderate fragrance), Chippendale (2 = good fragrance) and Blue River (3.5 = very strong fragrance) were used as a reference for the fragrance scores (see Suppl. Table S5 in Schulz et al. 2021). The distribution of the fragrance is shown in the box plots in Fig. 3.

### DNA extraction and quantification

DNA of the parental genotypes was extracted using the NucleoSpin ® Plant II Kit (MACHEREY–NAGEL GmbH & Co. KG, Düren, Germany) from young, unfolded leaves, which were stored for 24 h in the dark at room temperature. Afterwards 50 mg of frozen leaf tissue was homogenized in 2-mL test tubes with two steal beads using a bead mill (Retsch, Haan, Germany) at a frequency of 25 Hz for 2.5 min. The extracted DNA was quantified using a Nanodrop 2000c spectrophotometer (PeQLab Biotechnologie GmbH, Erlangen, Germany) and quality controlled in 1% agarose gels as described in Schulz et al. (2021).

### KASP assay for SNP-based selection of parental genotypes

KASP primers for the six analysed SNP markers Rh_PL_SNP49K, Rh_PN_SNP2K, Rh_PN_SNP6K, Rh_FR_SNP67K, Rh_FR_SNP139K and Rh_FR_SNP201K used to select parental genotypes are described in Schulz et al. (2021) and were designed by LGC Genomics (London, UK). Genotyping was performed using a QuantStudio 6 Real-Time PCR system (Applied Biosystems, USA) with 20-ng DNA, 5-µl KASP V4.0 Mastermix 384, High Rox (LGC Genomics, London, UK) and 0.14-µl KASP by Design Primer Mix in a final volume of 10 µl for each reaction. KASP thermocycling was performed as described by Schulz et al. (2021): activation for 15 min at 94 °C, followed by 10 cycles at 94 °C for 20 s and 61 °C for 1 min, decreasing by 0.6 °C per cycle), followed by 26 cycles at 94 °C for 20 s and 55 °C for 1 min. Reading of KASP genotyping reactions on the qPCR machine was performed in a final cycle at 30 °C for 30 s. Genotypic data were analysed using QuantStudio software v3.1 (Applied Biosystems, USA). Box plots of the segregation of all six KASP markers used in the 95-genotype association panel of Schulz et al. (2021) are shown in the supplementary figures.

### Statistical analysis

Normal distribution of the data was tested separately for each population using a Shapiro–Wilk test at a significance level of 0.05.The non-parametric Kruskal‒Wallis rank sum test was afterwards used to detect significant differences in SNP effects between cultivar groups for petal number and fragrance (*p* < 0.05; Dunn’s method). All pairwise multiple comparison procedures were used to identify significant differences in petal length using a two-sample *t* test (overall significance level = 0.05). Statistical calculations were performed in Excel 2016, MYSTAT 12 (Systat Software, Inc.) and QtiPlot 1.0.0.

## Results

A total of 384 parental genotypes from a commercial cut rose breeding programme were analysed with six KASP markers previously associated with either petal length, petal number or fragrance (Schulz et al. 2021, Table 1, Supplementary Fig. 1 to 6). The aim was to identify parents with either minimum or maximum allele dosages so that progeny resulting from crosses between extreme dosage groups could be compared phenotypically. Not all markers could be called for allele dosage in all traits, resulting in 324 to 373 dosage calls for the six markers.

**Table 1:** Allele dosages of markers analysed in cut rose genotypes for the selection of parental genotypes

Trait	Marker	Position	Allele dosage					Total genotypes
			0	1	2	3	4	
Petal length	Rh_PL_SNP49K	Chr 5, 14.50 Mbp	**77**	127	100	35	*5*	344
Petal number	Rh_PN_SNP2K	Chr 3, 33.56 Mbp	**1**	28	86	162	*93*	370
	Rh_PN_SNP6K	Chr 1, 53.20 Mbp	**110**	185	66	*1*	-	362
Fragrance	Rh_FR_SNP67K	Chr 2, 71.84 Mbp	**168**	65	71	17	*52*	373
	Rh_FR_SNP139K	Chr 2, 72.49 Mbp	*4*	103	114	72	**31**	324
	Rh_FR_SNP201K	Chr 3, 7.30 Mbp	**41**	104	149	56	*12*	362

The “Total genotypes” column shows the number of genotypes for which the respective KASP markers could be analysed out of a total of 384 genotypes. Allele dosages associated with reduced trait expression are shown in bold, while those associated with increased trait expression are shown in italics. The position refers to the *R. chinensis* genome from Hibrand Saint-Oyant et al. (2018).

Based on these SNP data, parents with contrasting allele dosages were selected for each of the three traits and crossed. For further analyses, only progenies with more than 10 individuals were used for phenotyping. Due to these limitations, the selected progeny did not always result from crosses of parents with the most extreme allele dosages of 0 or 4 but also included those with heterozygous dosages in the parents.

### Progeny segregating for petal length

Our previous GWAS in garden roses revealed large differences in petal length between genotypes with dosage 0 (34.8 mm) and dosage 4 (53 mm) for the SNP marker Rh_PL_SNP49K (Schulz et al. 2021). Based on the KASP analyses for this marker, eight different genotypes were selected as parents for the crosses with either high or low allele dosages and were expected to result in progeny with longer petals (long 1 and long 2) or shorter petals (short 1 and short 2) (Table 2).

**Table 2:** Crosses made on the basis of differences in petal length using SNP marker Rh_PL_SNP49K information to select parental genotypes

Cross name	Female × male parent	Allele dosage Rh_PL_SNP49K^a^	Number of individuals
Long 1	RT 12356 × Deep Water Water	3/3	16
Long 2	Lizzy × Revival	2/3	15
Short 1	Newsflash × Lampion	0/1	41
Short 2	Flashlight × Ozeana	0/0	41

^a^Allele dosage of the female/male parent for each cross. Effect of marker Rh_PL_SNP49K (chr. 5) in Schulz et al. (2021); see Suppl. Figure 1: dosage 0 = short petals, dosages 1 to 4 = long petals

The petal length was normally distributed in all four progenies. The two progenies of parental genotypes with favourable allele dosages (long1 and long2 with a dosage of 2 or 3) had significantly higher mean petal lengths (46.6 mm and 43.2 mm) than the progeny of parents with low allele dosages (short 1 and short 2) at 37.6 mm and 38.4 mm (Fig. 1). In addition, a combination of two triplex allele dosage genotypes for very long petals (long 1) resulted in an even higher mean petal length in the progeny than a cross of the duplex genotype ‘Lizzy’ and the triplex genotype ‘Revival’ (Fig. 1), although this difference was not statistically significant.

### Petal numbers

In the association study by Schulz et al. (2021), the marker Rh_PN_SNP2K was associated with petal number in an additive relationship between allele dosage and petal number (Suppl. Figure 2). In contrast, a second marker, Rh_PN_SNP6K, showed a dominant effect (Suppl. Figure 3). Genotypes with allele dosage 0 for Rh_PN_SNP6K had a mean value of 36.6 petals, and cultivars with dosage 1 or 2 had a mean value of 60.6 petals (Schulz et al. 2021). For both markers, the allele distribution among the cut rose parents was extremely skewed. While for the Rh_PN_SNP2K marker, only one genotype with dosage 0 was detected among 370 scored genotypes, the Rh_PN_SNP6K marker did not identify any genotypes with dosage 4 among 362 scored genotypes (Table 1). Therefore, the simultaneous selection of parental genotypes for both markers included cases with less extreme dosages for one of the two markers (Table 3).

**Table 3:** Crosses made for differences in petal number segregation using the SNP marker Rh_PN_SNP2K and Rh_PN_SNP6K information to select the parental genotypes

Cross name	Female × male parent	Allele dosage Rh_PN_SNP2K^a^	Allele dosage Rh_PN_SNP6K	Number of individuals
High 1	Silverado × RT04055	3/4	2/2	22
High 2	Gospel × Romina	3/4	0/1	14
High 3	Lizzy × Revival	4/4	0/1	15
Low 1	Syra × Eyes up	1/0	1/0	23
Low 2	Salsa × Eyes for you	1/1	0/0	11

^a^Allele dosage of female/male parent for each cross. For the effect of markers, see Schulz et al. (2021) and Suppl. Figure 2 and 3. Rh_PN_SNP2K (chr. 3): dosage 4 = high petal number; Rh_PN_SNP6K (chr. 1): dosage 0 = intermediately filled flowers (Ø 36.6 petals), dosages 1 and 2 = highly filled flowers (60.7 and 60.6 petals, respectively)

Although we were not able to select parents with the optimal allele configurations for both markers (Table 3), we observed highly significant differences in mean petal numbers between some of the progenies. Progenies from the crosses with the allele configurations associated with lower petal numbers also showed lower mean petal numbers, although not all pairwise high–low comparisons yielded significant differences (Fig. 2). When the progenies for the high- and low-petal-number allele dosages were analysed as one group each, the differences between the two groups were significant, with a mean of 52.6 petals for the “high” group versus 16.2 petals for the “low” group (Suppl. Figure 7). Only eight genotypes of the progeny from the low-petal-number crosses had the same or a higher petal number (≥ 16) compared with that of the genotypes with the lowest petal number from the high-petal-number crosses (high 1 to 3). Only the population high 2 showed a normal distribution of the petal number.

### Fragrance

The selection of parents with high and low allele dosages associated with fragrance was made using three SNP markers previously associated with fragrance in garden roses (Table 1, Schulz et al. 2021). Two of these markers (Rh_FR_SNP67K and Rh_FR_SNP139K) are located on chr. 2 and one (Rh_FR_SNP201K) on chr. 3.

Three parental combinations were selected for parental markers predicting strong fragrance (named strong 1– strong 3), and three crosses were selected for allele dosages predicting weak fragrance (named weak 1– weak 3). As with petal number, it was not possible to select only parents with optimal allele dosage combinations for all three markers simultaneously (Table 4). In particular, an allele configuration of 3 or 4 for Rh_FR_SNP201K would have been desirable in the weak 1– weak 3 crosses.

**Table 4:** Crosses made for differences in fragrance in the progenies using the SNP markers Rh_FR_SNP67K, Rh_FR_SNP139K and Rh_FR_SNP201K to select the parental genotypes

Cross name	Female × male parent	Allele dosage Rh_FR_SNP67K^a^	Allele dosage Rh_FR_SNP139K	Allele dosage Rh_FR_SNP201K	Progeny number
Strong 1	Rgrf. M Henriette × Romina	4/4	4/3	0/1	34
Strong 2	Rgrf. M Henriette × La Perla	4/3	4/3	0/2	15
Strong 3	Gospel × Romina	4/4	4/3	0/1	14
Weak 1	Sommersonne × Colossal Meidiland	0/0	0/1	3/2	26
Weak 2	G. Vancouver × Sommersonne	0/0	1/1	3/2	18
Weak 3	Cherry Girl × Gebrüder Grimm	0/0	2/2	1/1	15

^a^Allele dosage of the female/male parent for each cross. For the effect of markers in Schulz et al. (2021), see Suppl. Figures 4 to 6: Rh_FR_SNP67K (chr. 2), dosage 0 to 3 = weak fragrance, dosage 4 = strong fragrance; Rh_FR_SNP139K (chr. 2): dosage 0 = weak fragrance, dosage 4 = strong fragrance; Rh_FR_SNP201K (chr. 3): dosage 0 = strong fragrance, dosage 4 = weak fragrance

The analysis of scent in the individual progeny showed a large amount of variation, especially within the crosses for strong fragrance (Fig. 3). Only the strong 3 population showed a normal distribution of the fragrance data. The means of the strong crosses were all significantly higher than the means of the weak crosses. When individuals from all strong (*n* = 63) and all weak crosses (*n* = 59) were pooled, the means were highly significantly different (Suppl. Figure 8). Individuals with a fragrance score > 1.1 were observed only from the crosses of strong 1 and strong 3 and not from the 59 progeny of the combination weak 1 and weak 3.

## Discussion

Although the use of molecular markers in the breeding and production of rosaceous crops has enabled tremendous progress to be made in the last decade (Peace 2017), relatively little progress has been made in roses. The commercial use of markers has mainly been limited to genotyping to ensure intellectual property protection, while the development of markers associated with commercially important traits has been limited to academic research projects.

Here, we present data showing that markers for petal size, petal length and floral scent developed in previous studies (Schulz et al. 2021) successfully predict the outcome of these traits in commercial crosses. The markers had been identified in a previous GWAS in a small panel of 95 garden rose genotypes. Although larger panels are typically used in most GWASs in plants (Korte and Farlow 2013; Xingbo and Alexander 2020; Wang and Xu 2019; Tibbs Cortes et al. 2021), our original analysis in a small panel allowed us to detect some large-effect QTLs that could be validated in larger independent populations in this study. Here, we obtained further independent support for these marker‒trait associations by successfully predicting positive and negative effects using these markers in the progeny of crosses between parents selected for contrasting allele dosages.

Considering individual characters, not all crosses resulted in progeny with significantly contrasting phenotypes, as expected from the previous parental selection. Progeny scored for markers associated with petal length and fragrance were all significantly different, whereas individual progeny for markers associated with petal number did not show significant differences in all possible high vs. low marker comparisons. However, the combination of families showed significant differences between the combined high-petal-number group and the low-petal-number group. There are several reasons why a clear difference between all families may be obscured. First, the fact that only a single flower was scored for each individual, whereas multiple flowers from three clones of each genotype were scored for the original GWAS, resulted in lower precision of phenotyping here. In addition, the family sizes were small due to the limitations of the populations available. The large variation within families scored for scent is most likely because it was not possible to select only parents with the optimal allele dosage combinations for the three markers. However, the variation could also be due to the nature of the scoring scheme, with only one flower per genotype tested.

Also important for future applications of these markers is the observation that although these markers were originally identified in a GWAS panel of garden roses, they also show high predictive power in cut roses. Cut roses are by far the most economically important group of roses and are bred in programmes separate from those of garden and pot roses (Debener and Byrne 2014; De Vries and Dubois 2020). Although the genetic diversity of cut roses appears to be lower than that of garden roses (Vukosavljev et al. 2013), we were able to show that marker information can be easily transferred between the two groups/gene pools. Furthermore, it also reflects the similar genetic architectures of these flower traits in the two gene pools, with similar alleles acting on the phenotypes. Whether this can be extended to other traits needs to be investigated in the future.

As the floriculture market grows from approximately US$7 billion in 2018 to an expected value of more than US$11 billion in 2027 for cut flowers alone, the demand for new innovative genotypes with elite characteristics will increase (Giovannini et al. 2021). This is in contrast to the selection methods currently used by most rose breeding companies, which are associated with high effort to release new varieties. Commercial rose breeding is mainly based on large progenies of several hundred thousand seedlings selected only by phenotypic screens. Parental plants are also selected only for harmonising phenotypic and productivity traits (Crespel and Mouchotte 2003) based on records kept by breeders. Only 1–3% of the progeny are selected for easily scored and highly heritable traits (such as petal number and size) at the early seedling stage (Leus et al. 2018).

Given this situation, direct selection of seedlings based on marker data seems rather unrealistic in commercial rose breeding for which no examples of marker-assisted selection has been made public so far. In contrast, selection from the much smaller population of parental genotypes using marker information is a more feasible alternative requiring less resources. Nonetheless, it remains important to consider that the desired traits and their inheritance must be carefully evaluated even in marker-assisted parental selection. Trait selection should prioritize characteristics that cannot be efficiently improved using standard breeding and selection methods. Traits that can only be evaluated with large plant numbers at later selection cycles, such as productivity or resistance traits, should also be prioritized (Smulders and Arens 2018). Additionally, it is essential to consider the heritability and environmental factors that influence the trait expression.

Although phenotyping of the three floral traits analysed here is straightforward, marker-informed selection of parental plants is effective if it is performed with additional information on genotype and not only phenotype, as cut roses are highly heterozygous tetraploids (Smulders and Arens 2018). In autotetraploid crops, genotypes that are heterozygous for a given locus exhibit three types of allele dosages (simplex, duplex and triplex), which result in different segregation ratios in the progeny of crosses (Smulders et al. 2019). The higher the dosage of favourable alleles in both parents is, the higher the proportion of progeny with increased expression of the favoured phenotype, which can be expected. Furthermore, some marker‒trait associations follow an additive model, so in addition to the fraction of progeny with favourable alleles, the expected progeny with the favourable allele dosage could also be increased by the selection of suitable parents.

To ensure at least the same probability of identifying seedlings with new superior traits, this reduced number of parental genotypes needs to be selected more carefully (Witcombe et al. 2013). However, if overall population sizes are maintained, selection intensity for other traits could be increased, or traits with high heritability (e.g. disease resistance) could already be included in the selection of seedling populations.

If parental selection is used, it is important to ensure that the genetic diversity in the cut rose breeding pool is not further reduced. Molecular markers may also be an appropriate tool for maintaining genetic diversity in this gene pool.

## Conclusions

We have shown that markers associated with three floral traits in garden roses are suitable for marker-assisted selection in cut rose breeding based on selecting parents with favourable allele dosages.

Improving the efficiency of selection in cut rose breeding by MAS of parental genotypes could be very important in the future to adapt important flower traits for changing markets within shorter breeding cycles. An example of this is the trend towards smaller flower sizes with fewer and shorter petals in Europe due to the sale of cut roses in supermarkets and their longer vase life.

### Supplementary Information

Below is the link to the electronic supplementary material.

Supplementary file1 (DOCX 407 KB)

## Data Availability

The datasets generated during and/or analysed during the current study are available from the corresponding author on reasonable request.
