# Supplementary material for: Robust markers associated with floral traits in roses are suitable for marker-assisted selection across gene pools
Source: Mol Breed. 2023 Dec 8;43(12):90. doi: 10.1007/s11032-023-01438-5 (PMC10709285; doi:10.1007/s11032-023-01438-5)
Supplement: Supplementary file 1 — Supplementary file1 (DOCX 407 KB) [file 11032_2023_1438_MOESM1_ESM.docx]

**Supplementary material:**

**Supplementary Fig. S1** SNP marker Rh_PL_SNP49K on chr. 5 for petal analysed in 95 cultivars of the association panel. On the x-axis are the allelic dosages for the four marker classes (0 and 4 for the homozygous classes and 1-3 for the heterozygotes) and on the y-axis the petal length is shown. Significant differences between the means of two groups are indicated by different letters below the boxes (Kruskal-Wallis analysis of variance, Dunn's post-hoc test; p = <0.05). For each progeny, the number of individuals (n) is given below the boxes; mean values are shown left above the box.

**Supplementary Fig. S2** SNP marker Rh_PN_SNP2K on chr. 3 for petal number with 95 cultivars in the association panel. On the x-axis are the allelic dosages for the four marker classes (0 and 4 for the homozygous classes and 1-3 for the heterozygotes) and on the y-axis the petal number is shown. Above the box plots the number of individuals scored per allele dosage are given. Letters above the whiskers describe mark groups that are significantly different at p<=0.05 (Tukey Kramer post-hoc test).

**Supplementary Fig. S3** SNP marker Rh_PN_SNP6K on Chr. 1 for petal number with 95 cultivars in the association panel. On the x-axis are the allelic dosages for the three observed marker classes (the homozygous class 0 and the heterozygous classes 1 and 2) and on the y-axis the petal number is shown. Above the box plots the number of individuals scored per allele dosage are given. Letters above the whiskers describe mark groups that are significantly different at p<=0.05 (Tukey Kramer post-hoc test).

**Supplementary Fig. S4** Validation of the SNP marker Rh_FR_SNP67K for fragrance with 94 cultivars in the association panel. On the x-axis are the allelic dosages for the four marker classes (0 and 4 for the homozygote classes and 1-3 for the heterozygotes) and on the y-axis the fragrance. Shown above the box plots are the number of individuals scored. Anova with Games Howell post-hoc test.

**Supplementary Fig. S5** Validation of the SNP marker Rh_FR_SNP139K for fragrance with 96 cultivars in the association panel. On the x-axis are the allelic dosages for the four marker classes (0 and 4 for the homozygote classes and 1-3 for the heterozygotes) and on the y-axis the fragrance. Shown above the box plots are the number of individuals scored. Anova with Games Howell post-hoc test.

**Supplementary Fig. S6** Validation of the SNP marker Rh_FR_SNP201K for fragrance with 95 cultivars in the association panel. On the x-axis are the allelic dosages for the four marker classes (0 and 4 for the homozygote classes and 1-3 for the heterozygotes) and on the y-axis the fragrance. Shown above the box plots are the number of individuals scored. Anova with Games Howell post-hoc test.

**
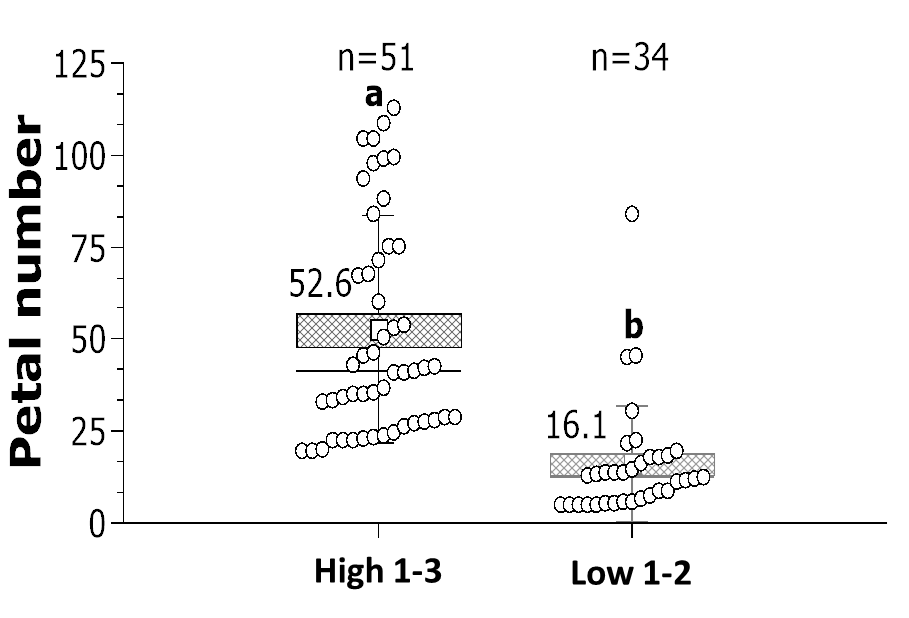
**

**Supplementary Fig. S7:** Box plots for mean petal number of progeny from five crosses with contrasting allele dosages. Progeny for the high and low petal number allele dosages were analysed as one group each. Significant differences between the means of the two pooled groups are indicated by different letters above the boxes (Mann-Whitney Rank Sum Test p=<0.001). For each progeny, the number of individuals (n) is given above the boxes; mean values are shown left above the box.


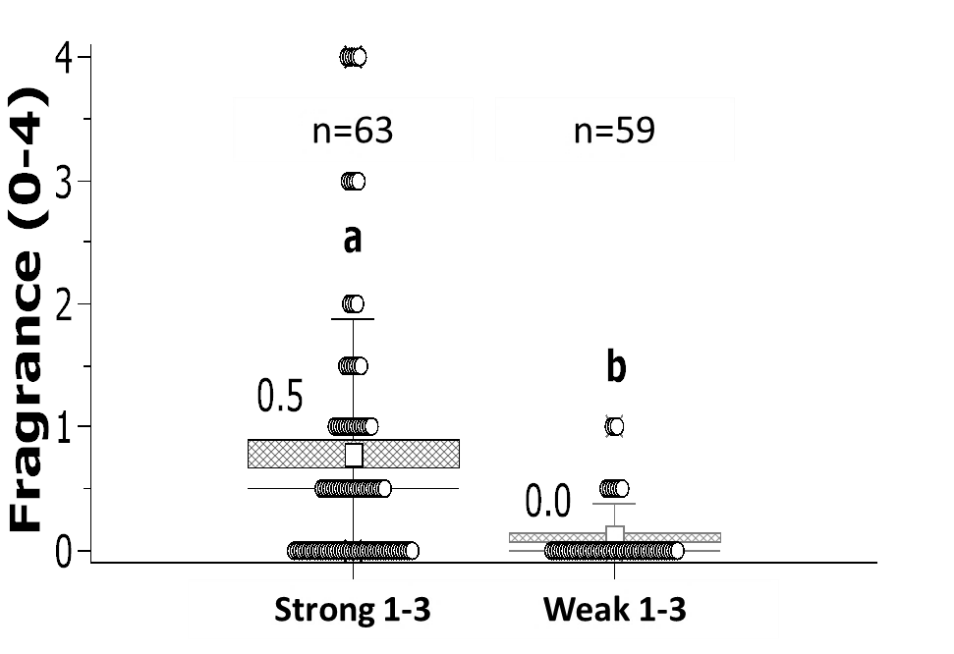


**Supplementary Fig. S8**: Significant difference in populations for strong vs. weak fragrance, respectively. The progeny of the four strong scent crosses and the three weak scent crosses are each merged into a group. Significant differences between the means of the two pooled groups are indicated by different letters above the boxes (Mann-Whitney Rank Sum Test p=<0.001). For each progeny, the number of individuals (n) is given above the boxes; mean values are shown left above the box.
